# Mesenchymal stem cells secretome as a modulator of the neurogenic niche: basic insights and therapeutic opportunities

**DOI:** 10.3389/fncel.2015.00249

**Published:** 2015-07-13

**Authors:** Antonio J. Salgado, Joao C. Sousa, Bruno M. Costa, Ana O. Pires, António Mateus-Pinheiro, F. G. Teixeira, Luisa Pinto, Nuno Sousa

**Affiliations:** ^1^Life and Health Sciences Research Institute (ICVS), School of Health Sciences, University of MinhoBraga, Portugal; ^2^ICVS/3B’s, PT Government Associate LaboratoryBraga/Guimarães, Portugal

**Keywords:** mesenchymal stem cells, neural stem cells, niche, neurogenesis, secretome, regenerative medicine, interactions

## Abstract

Neural stem cells (NSCs) and mesenchymal stem cells (MSCs) share few characteristics apart from self-renewal and multipotency. In fact, the neurogenic and osteogenic stem cell niches derive from two distinct embryonary structures; while the later originates from the mesoderm, as all the connective tissues do, the first derives from the ectoderm. Therefore, it is highly unlikely that stem cells isolated from one niche could form terminally differentiated cells from the other. Additionally, these two niches are associated to tissues/systems (e.g., bone and central nervous system) that have markedly different needs and display diverse functions within the human body. Nevertheless they do share common features. For instance, the differentiation of both NSCs and MSCs is intimately associated with the bone morphogenetic protein family. Moreover, both NSCs and MSCs secrete a panel of common growth factors, such as nerve growth factor (NGF), glial derived neurotrophic factor (GDNF), and brain derived neurotrophic factor (BDNF), among others. But it is not the features they share but the interaction between them that seem most important, and worth exploring; namely, it has already been shown that there are mutually beneficially effects when these cell types are co-cultured *in vitro*. In fact the use of MSCs, and their secretome, become a strong candidate to be used as a therapeutic tool for CNS applications, namely by triggering the endogenous proliferation and differentiation of neural progenitors, among other mechanisms. Quite interestingly it was recently revealed that MSCs could be found in the human brain, in the vicinity of capillaries. In the present review we highlight how MSCs and NSCs in the neurogenic niches interact. Furthermore, we propose directions on this field and explore the future therapeutic possibilities that may arise from the combination/interaction of MSCs and NSCs.

## Introduction

Injury and disease within the central nervous system (CNS) frequently induce chronic and acute insults leading to irreversible processes of neuronal cell death. Understanding how neurogenesis can be modulated, either through drugs or interaction with other cell types, and neural progenitors recruited to the site of injury, is of the utmost importance for the development of novel strategies that may impact the current state of the art. In recent years it has become evident that a population with a non-neural phenotype known for their role in the osteogenic niche, mesenchymal stem cells (MSCs), is able to regulate important phenomena within the CNS, including neural progenitor cells proliferation and differentiation. This quite unexpected and surprising function of MSCs brought closer the neurogenic and osteogenic niches, and prompted a new field of research that aims at understanding their interaction, and how both may impact on CNS regenerative medicine as we know it. Having this in mind the objective of the present paper is to review the most relevant advances in this field. It will first give an overview of neurogenic niches and how neurogenesis is regulated within them, then give an introduction to the osteogenic niches and MSCs, and end with a review on the most important works on the interactions between MSCs, neurogenic niches and disease models within the CNS.

## Neurogenesis in the Adult Brain

Neuroanatomists have long believed Cajal’s assumptions on the immutability of the CNS. This dogma has been challenged due to growing evidence that endow the brain with considerable regenerative potential and neuroplastic capacity, essential to promote brain homeostasis (Lemaire et al., 2012). It is now well established that adult neurogenesis occurs throughout life in specific brain regions where neurons are constantly generated (Doetsch et al., 1999; Gage, 2002).

**Figure 1:**
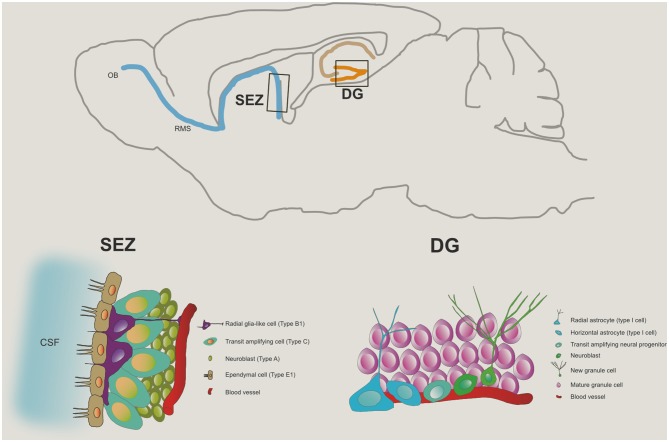
**Neurogenic niches in the adult brain**. The top panel represents, in a saggital section of the rodent brain, the two major niches of neural progenitor cells in the adult brain: one, in the sub granular zone of the dentate gyrus (DG) of the hippocampus, and the other, the subependymal zone (SEZ), from where progenitor cells committed to the neuronal lineage migrate via the rostral migratory stream (RMS) towards the olfactory bulb (OB). The bottom left panel illustrates the typical cytoarchitecture of the SEZ niche while the cell population in the DG niche is presented in the bottom right panel.

Globally, this neuroadaptative phenomenon occurs by the re-organization of the neuromorphological and electrophysiological properties of post-mitotic cells and the generation of new neuronal or glial cells that will incorporate the pre-existing networks, a process therefore called neuro- or gliogenesis, respectively (Guan et al., 2009). This complex process involves several steps beyond cell division; these include the commitment of the new cell to a neuronal phenotype, the migration and morphophysiological maturation of the neuroblasts, and the establishment of appropriate synaptic contacts that culminate with a full integration on the pre-existent network. These spatially defined brain regions where neurogenesis occurs display a permissive microenvironment for maintenance, proliferation and differentiation of Neural stem cells (NSCs). Admittedly, at least two defined neurogenic brain regions are broadly recognized in the adult mammalian brain (Figure 1): the subependymal zone (SEZ) of the lateral ventricles, and the subgranular zone (SGZ) of the hippocampal dentate gyrus (DG; Zhao et al., 2008). In both regions, astroglial cells act as the source of adult progenitor cells (Seri et al., 2001).

In the adult hippocampal neurogenic region, the progenitor cells reside in the SGZ, with defined gradients (Silva et al., 2006). Newly-born cells generated in the SGZ, become committed to a neuronal lineage and migrate into the granular cell layer (GCL), where they mature to become glutamatergic granule neurons (Seri et al., 2004; Zhao et al., 2008; Brill et al., 2009). The neuroblasts born in the SEZ migrate anteriorly along the rostral migratory stream (RMS), becoming mostly mature GABAergic granule neurons and periglomerular interneurons in the olfactory bulb (OB; Chumley et al., 2007; Zhao et al., 2008). Besides these two well-accepted adult neurogenic regions, although disputable, some reports have shown evidences for the generation of new neurons on other brain regions of the adult brain, including the amygdala (Bernier et al., 2002; Fowler et al., 2005; Gonçalves et al., 2008), the hypothalamus (Fowler et al., 2002; Kokoeva et al., 2005), the cortex (Gould et al., 1999; Kodama et al., 2004), the striatum (Dayer et al., 2005; Bédard et al., 2006) and the substantia nigra (SN; Zhao et al., 2003; Yoshimi et al., 2005). Importantly, it appears that neurogenesis in these regions occurs at very low levels or under non-physiological conditions (von Bohlen und Halbach, 2011).

Importantly, the neurogenesis process in the adult brain constitutes a new dimension of plasticity, with great impact on neuronal remodeling and repair, being now considered by the biomedical field as a promising therapeutical target in several neuropathological contexts. For instance, abnormal alterations in the hippocampal neurogenesis process have been implicated in an assortment of neuropsychiatric disorders (Sapolsky, 2000; Eisch et al., 2008; Kobayashi, 2009). Indeed, impairments in neuroplasticity are increasingly considered central to the ethiopathogenesis of depression (Bessa et al., 2009; Mateus-Pinheiro et al., 2013a,b). Studies have also shown the contribution of new neurons to a subset of hippocampal functions, influencing mood control, learning and memory (Hanson et al., 2011; Eisch and Petrik, 2012; Konefal et al., 2013). In fact, a clear connection between adult neurogenesis and learning/memory was demonstrated, as diminished neurogenesis decreases learning/memory, while enhanced neurogenesis improves it (Eisch and Petrik, 2012; Nakashiba et al., 2012). These examples prompt for the relevance of modulating the neurogenic niches as a potential therapeutic strategy to treat the symptoms of neurodegenerative disorders such as Parkinson’s disease (PD), which we will later develop in the context of MSCs derived therapies.

We will next refer to the structural and functional organization specificities of the adult SGZ and SEZ neurogenic niches.

### Adult Hippocampal Neurogenesis

As referred above, the adult brain is capable of generating new cells that can incorporate into its established complex circuitry (Trujillo et al., 2009). This process of adult neurogenesis highly recapitulates the embryonic neurogenic process, with the important difference that new neurons are generated in an already mature microenvironment and have to integrate in pre-existing neural circuits. Adult hippocampal neurogenesis consists of several highly regulated sequential phases (Kempermann et al., 2004; Ming and Song, 2005) characterized by morphological distinct cells: (i) proliferation of neural progenitor cells residing in a narrow layer of about three nuclei wide, the SGZ; (ii) generation of amplifying progenitors; (iii) cell migration; (iv) differentiation; and (v) maturation at the final destination with axon and dendrites formation and establishment of new synapses (Kempermann et al., 2004; Steiner et al., 2006; Balu and Lucki, 2009).

The adult SGZ contains heterogeneous progenitor cells, which can be distinguished and identified by a particular set of molecules expressed by each progenitor population. The first type of progenitors are the quiescent neural progenitors (QNPs), described to be multipotent stem cells (Seri et al., 2001, 2004) and also known as NSCs or type-1 progenitor cells (Type-1 cells). These cells have morphological and antigenic glial properties, expressing markers such as the intermediate filament protein nestin, brain lipid binding-protein (BLPB), the glutamate aspartate transporter (GLAST; Steiner et al., 2006) and glial fibrillary acidic protein (GFAP), among others; it can be further distinguishable into two subtypes, based on their spatial orientation in the SGZ: radial astrocytes (rA) and horizontal astrocytes (hA). Radial astrocytes are characterized by having a single radial process, being also slowly dividing cells, whereas hA present a short horizontal process and divide faster (Lugert et al., 2010; Hodge et al., 2012). These cells divide asymmetrically giving rise to transient amplifying neural progenitors (tANPs, also designated as type-2 progenitor cells or TAPs). It is important to notice that this phase of the neurogenic process comprises a decisive point in the determination of neural progenitors cell-fate (neuronal or non-neuronal lineage commitment; Steiner et al., 2006). This latter progenitor cells, TAPs, are already committed to a neuronal lineage, being mitotically active (Encinas et al., 2006) and dividing symmetrically to give rise to neuroblasts (also known as type-3 cells). Neuroblasts are intermediate progenitors in the generation of new glutamatergic granule neurons, corresponding to a stage of transition from a slowly proliferating neuroblast, which is exiting the cell cycle, to a postmitotic immature neuron, that will migrate into the GCL of the DG. These neuroblasts express markers of the neuronal lineage, such as the polysialylated-neural cell adhesion molecule (PSA-NCAM), calcium-binding protein calretinin and doublecortin (DCX), that are crucial for further maturation and migration of these cells (Pleasure et al., 2000; Ehninger and Kempermann, 2003; Balu and Lucki, 2009). When reaching the GCL, newborn cells will fully maturate, elongating their axons towards the CA3 region (von Bohlen und Halbach, 2011) and establishing new functional connections (Balu and Lucki, 2009), thus becoming mature granule neurons, which express neuronal nuclei protein (NeuN). The cell markers described above are not all exclusive to the SGZ; as will be described next, some are also characteristic of cells from the SEZ niche (Table 1). Moreover, similarly to the SEZ, only some of these markers allow cell-specific phenotypic characterization, as indicated in Table 1. Approximately 2–3 weeks after exiting the cell cycle, they express calbindin, a marker of mature granule cells (Kempermann et al., 2004). Newly formed neurons enter a period of enhanced synaptic plasticity in which their electrophysiological properties resemble those of neurons in the early postnatal period in juvenile animals (Ge et al., 2007). This phase lasts around 4–6 weeks after the original cell division, resulting in a total of approximately 7–8 weeks required for newborn cells to become functionally indistinguishable from the older granule cell population (Carlén et al., 2002; Abrous et al., 2005; Zhao et al., 2008; Snyder et al., 2009; Hanson et al., 2011). Newborn neurons display very different characteristics than mature ones, such as enhanced excitability, reduced threshold to induction of long-term potentiation (LTP) and an excitatory response to GABAergic input, since this neurotransmitter induces depolarization instead of hyperpolarization that is seen in adult neurons, which is related to a specific pattern of expression of some ionic co-transporters. This clearly indicates that adult-born neurons possess specific properties associated with plasticity (Schmidt-Hieber et al., 2004; Saxe et al., 2006; Ge et al., 2007; Hanson et al., 2011).

**Table 1:** **Summary of markers that specifically allow phenotypic characterization of major cell types found in both neurogenic and osteogenic niches**.

		Type-1 (NSCs)	Type-2 (TAPs)	Type-3 (Neuroblasts)	Mature neurons
**Neurogenic niches**	**SGZ**	GFAP	Mash1	DCX	NeuN
		GLAST	Tbr2	PSA-NCAM
			Ngn2
		**Type B (NSCs)**	**Type C (TAPs)**	**Type A (Neuroblasts)**	**Mature neurons**
	**SEZ**	GFAP	Mash1	DCX	NeuN
		GLAST	Dlx2	PSA-NCAM	Calretinin
					Calbidin
					GAD65
					TH
**Osteogenic niches**	**Osteoblasts**	Runx-2; OCN; OPN; ON; ALP		
	**MSCs**	Positive for CD105, CD73, CD90 Negative for CD45, CD34, CD14, CD11b, CD79a, CD19		

*SGZ, subgranular zone; SEZ, subependymal zone; NSCs, neural stem cells; TAPs, transient amplifying progenitors; GFAP, glial fibrillary acidic protein; GLAST, glutamate aspartate transporter; Mash1, mammalian achaete-scute complex homolog 1; Tbr2, T-box brain 2; Ngn2, neurogenin 2; DCX, doublecortin; PSA-NCAM, polysialylated-neural cell adhesion molecule; NeuN, neuronal nuclei; Dlx2, distal-less homeobox 2; GAD65, glutamate decarboxylase 65; TH, tyrosine hydroxylase; OCN, osteocalcin; OPN, osteopontin; ON, osteonectin; ALP, alkaline phosphatase*.

Noticeably, neurogenesis is a fine tuned process, in which not all cells expressing immature neuronal markers develop into fully mature neurons (Kempermann et al., 2003) and most newly-born neurons are eliminated by apoptosis (Biebl et al., 2000). The mechanisms that regulate this clearance of neurons are still to be fully understood, however, very recently a report showed that DCX-neuronal progenitors present phagocytic activity in the hippocampal and SEZ neurogenic niches and have great impact in the neurogenic process (Lu et al., 2011).

The harmonization of the several processes and cellular activities that occurs during the generation of new neurons in the adult mammalian brain is thus paramount. Several studies propose a complex transcriptional and epigenetic orchestration of the adult hippocampal neurogenic process, with both intrinsic and extrinsic factors being ultimately responsible for the modulation of this phenomenon. Therefore, the niche, where adult neurogenesis occurs is also crucial for the modulation and fine-tuning of this process.

### Subependymal Zone Neurogenesis

The SEZ, also referred in the literature as adult subventricular zone (SVZ), is the site of the adult brain where neurogenesis is most intense. In rodents, the SEZ is seldom described as a thin layer of cells located below the ependymal layer that lines the lateral walls of the lateral ventricles, but it also extends to the dorsal and medial ventricular walls (Alvarez-Buylla et al., 2008). As in the SGZ niche, the cell populations in the SEZ are heterogeneous, containing several cell types that are identifiable by cell-specific markers. In general terms it might be described as being composed of slow-dividing type B cells (the NSCs) that originate fast-dividing type C cells, that in turn give rise to neuroblasts (type A cells). Nevertheless, given the complexity of these cell populations they, and respective phenotypic markers, will next be described with further detail (see also Table 1).

Type B cells are astrocytic cells and express the intermediate filament GFAP. In the SEZ two types of GFAP positive cells were distinguished according to ultrastructural differences: type B2 astrocytes, or niche astrocytes, display a highly branched morphology and are frequently found in the interface of the SEZ and the striatum (Doetsch et al., 1997); type B1 astrocytes are radial-glia like that organize in pinwheel structures with the apical ending, the primary cilium, turned towards the brain ventricles—and hence in bathed in the cerebrospinal fluid—and is surrounded by ependymal cells (Mirzadeh et al., 2008). The type B1 cells are recognized as the NSCs of the SEZ. Type C cells, or TAPs, originate from the NSCs. These rapidly dividing cells are organized in clusters of immature precursors that express distal-less homeobox 2 (Dlx2), achaete-scute complex homolog 1 (Ascl1or Mash1) and epidermal growth factor receptor (EFGR; Ciccolini et al., 2005; Ming and Song, 2011). A short pulse (24 h) of the timidine analog BrdU mainly labels TAPs indicating that these cells are the largest pool of proliferating cells in the SEZ. Type A cells, or neuroblasts, are born from type C cells and constitute the neuronal precursors cells. Most type A cells express PSA-NCAM and DCX, which are associated to their migratory properties (Ming and Song, 2011). Under physiological conditions neuroblasts migrate tangentially from the SEZ, via the RMS to the OBs where they become fully mature neurons. Neuroblasts divide actively in the SEZ but also in the RMS. Once in the OBs, neuroblasts migrate radially, give rise to mature neurons and are integrated in distinct layers of the OB. They form new granular cells (deep, superficial and calretin positive) and periglomerular cells (calretin positive, calbidin positive and tyrosine hydroxylase positive; Lledo et al., 2008; Kriegstein and Alvarez-Buylla, 2009). Most of these new neurons are granule cells integrated in the granule cell layer and are GABAergic, but a small group of glutamatergic neurons was also identified (Brill et al., 2009).

Also of relevance in the SEZ are the ependymal cells (type E cells) that, as indicated above, form a monolayer that outlines the ventricular wall. These cells constitute a physical barrier that diminishes the direct and free exchange of molecules between the CSF and brain parenchyma (Falcão et al., 2012a). Two distinct ependymal cells have been described: the most common type E1 ependymal cells that are multiciliated, and the E2 ependymal cells that display two long cilia and represent solely 5% of the type E cells (Mirzadeh et al., 2008). Under physiological conditions these cells proliferate rarely (Coskun et al., 2008) or do not proliferate at all (Mirzadeh et al., 2008).

Tanycytes (Doetsch et al., 1997; Chojnacki et al., 2009), microglia (in response to injury; Ekdahl et al., 2009) and endothelial cells of the blood vessels (Tavazoie et al., 2008) are also relevant cellular components of the SEZ niche. These later cell types contribute to the specific microenvironment that constitute the SEZ NSCs niche; for instance, endothelial cells secrete several factors (pigment epithelium-derived factor, PEDF; NT3, among others) that induce proliferation and migration of NSCs (Ramírez-Castillejo et al., 2006; Delgado et al., 2014). Hence their interaction with proliferating cells should be taken into account when considering the modulation of the SEZ NSCs namely if one targets, for neuroregenerative purposes, the application of exogenous cells and/or protein/molecular factors, as will be further discussed in later sections.

In addition to the SEZ cellular heterogeneity, there is a further level of complexity in the form of topographical heterogeneity. A simple observation on the topography of the SEZ discloses major anatomical differences (Falcão et al., 2012b). It is now evident that even in the above described cell populations lays a remarkable heterogeneity either due to inherited intrinsic or epigenetic factors (Alvarez-Buylla et al., 2008) and/or an additional diversity in the surrounding microenvironment cues. Several studies showed that the NSCs pool is highly heterogeneous both in the origin and in cellular fate (Merkle et al., 2007; Alvarez-Buylla et al., 2008). For instance, while the common fate of SEZ born cells is the OB where they become interneurons, it was shown that it also generates a small pool of glutamatergic neurons steming from NSCs that reside in the adult dorsal wall of the lateral (Brill et al., 2009). Moreover, neuroblasts born either in ventral, dorsal, anterior or posterior regions are distinct, produce different neuronal types and are integrated in different layers of the OB (Alvarez-Buylla et al., 2008). As an example, neuroblasts from dorsal regions mostly originate superficial granule cells; while ventral derived neuroblasts give rise mostly to deep granule cells (Merkle et al., 2007). Also of notice, SEZ NSCs also originate oligodendrocyte precursors that migrate to the striatum and the corpus callosum and differentiate into oligodendrocytes (Nait-Oumesmar et al., 1999; Picard-Riera et al., 2002). The reason for why different regionally placed NSCs give rise to distinct progeny might reside in the distribution pattern of specific transcription factors, adding another layer of complexity in the regulation of cell proliferation in the SEZ, and thus in cell fate. All of these cell intrinsic and extrinsic aspects must be taken into account when considering putative therapeutic approaches for CNS regeneration.

### Transcriptional Regulation of Adult Neurogenesis

Adult neurogenesis gives rise to both glutamatergic and GABAergic neurons. In the hippocampus changes in the rates of generation of glutamatergic neurons might contribute to several pathologies. In this context, the discovery of new factors important for the generation of glutamatergic neurons is needed. Interestingly, adult glutamatergic neurogenesis recapitulates the sequential expression of transcription factors found in the developing cerebral cortex (Pax6→Neurogenin2→Tbr2→Tbr1), demonstrating that this transcription network is maintained postnatally (Brill et al., 2009). For example, Pax6, a crucial determinant for the specification of glutamatergic neurons during development, is essential for adult neurogenesis (Hack et al., 2005) and is sufficient to instruct postnatal neocortical astrocytes towards neurogenesis *in vitro* (Heins et al., 2002). It was also shown during development, that one of the downstream targets of Pax6, the transcription factor AP2γ, is important for the specification of glutamatergic neocortical neurons and their progenitors (Pinto et al., 2009), and also for the differentiation of glutamatergic neurons in the adult neurogenic regions. Furthermore, AP2γ regulates Tbr2, which was shown to be important for glutamatergic neurogenesis during development (Pinto et al., 2009).

As described above, generation of specific cell types (neuronal or glial type) in the adult SEZ is topographically heterogeneous and this might be bound to transcriptional regulation. In fact, the expression of distinct transcription factors in both overlapping and non-overlapping regions of the SEZ is described. Similarly to the SGZ, some of these transcription factors were correlated with the SEZ embryonic origin (Waclaw et al., 2006; Young et al., 2007). In fact, a topographical pattern of transcription factors expression in the SEZ is associated with NSCs embryonic origin and adult neuronal fate. Generally, NSCs in the lateral ventricular wall ubiquitously express Dlx1, 2, 5 and Mash1, while Emx1 expression is exclusive to the dorsal wall of the ventricle (Young et al., 2007). Furthermore, the transcription factors Nkx2.1 and Pax6 outline the ventral and dorsal regions of the lateral wall, respectively (Alvarez-Buylla et al., 2008; Weinandy et al., 2011). Thus, in the SEZ, an additional challenge is to understand how to modulate different combinations of transcription factors so as to result in production of specific neuronal types.

A targeted induction of neurogenesis, by stimulating endogenous neural progenitors in the adult brain, could represent an important cellular therapy to treat neurodegenerative disorders. A major challenge in our days is to improve survival and induce differentiation of newborn neurons after acute lesions. For instance, it was already shown that Pax6 can induce neurogenesis from non-neurogenic astrocytes *in vivo*, when overexpressed after stab-wound lesion (Buffo et al., 2005). These experiments provide proof of principle that neurons can be newly generated from endogenous sources of the adult mammalian brain. However, these induced neurons are very few in number and fail to mature. Therefore, new cues are needed to efficiently instruct neurogenesis and repair after neuronal insult.

### The Microenvironment of the Neurogenic Niches

The interplay between extrinsic and intrinsic factors determines the NSCs niche homeostasis. Intrinsic factors are a set of signals produced by the progenitors that together with exterior microenvironment cues (extrinsic factors) instruct distinct neurogenic phases and ultimately the cellular fate. Many of the mechanisms regulating NSCs proliferation and neurogenesis during embryonic development, appear to be conserved in adulthood, and both intrinsic and extrinsic factors important for embryonic neurogenesis are also involved in the regulation of neurogenesis in the adult brain (Ming and Song, 2011). However, there are relevant differences between them, especially regarding the properties of the cellular and molecular niche. Whereas during development, the cellular environment is highly specialized to support proliferation, in the adult neurogenic niches the environmental context is concomitantly able to maintain a population of fully mature neurons (Zhao et al., 2008; Jessberger et al., 2009), thus providing a different set of both intrinsic and extrinsic signals.

Extrinsic signals, for instance, for the SEZ regulation include several trophic and growth factors, neurotransmitters, morphogens, hormones and cytokines (Falcão et al., 2012a). These extracellular signaling molecules are of diverse origins, namely from ependymal cells, endothelial cells, neural progenitor cells and neurons. The neurotransmitters are examples of key extrinsic factors of neuronal origin. For instance, the neurotransmitter GABA produced by niche neuroblasts is reported to inhibit NSCs proliferation but serotonine stimulates NSCs proliferation (Banasr et al., 2004, and conflicting results were presented for the effects of dopamine (DA) in the SEZ niche (Berg et al., 2013).

This important role of the microenvironment in the neurogenic niches for the regulation of NSCs has been shown by many *in vivo* and *in vitro* studies. For example, SEZ derived neuroblasts can change their fate and differentiate into oligodendrocytes upon a change in the microenvironment induced by demyelination of the corpus callosum (Picard-Riera et al., 2002; Jablonska et al., 2010). Additionally, glial progenitor cells may change to a neuronal fate when transplanted into a neurogenic region (Shihabuddin et al., 2000), while mouse SEZ neural progenitors committed to the neuronal lineage, changed to glial differentiation upon transplantation into regions outside the neurogenic niche (Seidenfaden et al., 2006).

The microenvironment of the neurogenic niches is thus essential for fate determination and cell differentiation, as well as for self-renewal, proliferation, migration and maturation of NSCs. This microenvironment is comprised of local cell types, cell signals, extracellular matrix and microvasculature. Indeed, the SEZ and SGZ niches are highly vascularized by a network of specialized capillaries (Goldberg and Hirschi, 2009) and NSCs closely interact with the microvasculature (Palmer et al., 2000; Mirzadeh et al., 2008; Shen et al., 2008; Tavazoie et al., 2008). This microvasculature has been shown to be essential in maintaining the function of the neurogenic niches, namely by regulating the proliferation and quiescence of NSCs (Palmer et al., 2000; Shen et al., 2004, 2008; Tavazoie et al., 2008; Culver et al., 2013), as well as NSCs self-renewal and neurogenesis through soluble factors secreted by the endothelial cells (Shen et al., 2004; Ramírez-Castillejo et al., 2006; Gómez-Gaviro et al., 2012). Noteworthy is the recent report of the existence of MSCs in the brain microvasculature (Paul et al., 2012), which paves way for the usage of MSCs secretome to modulate the neurogenic niches cells. One further example of NSCs microenvironment modulators are microglia cells, the brain resident macrophages, have also a crucial role in the regulation and maintenance of neurogenesis in the SGZ neurogenic niche (Sierra et al., 2010) given that they impact on the proliferation of neural stem/progenitor cells (Gebara et al., 2013); also they are particularly relevant in modulating the SEZ in response to brain injury (Thored et al., 2009).

In this way, signaling from and into the niche is suggested to be responsible for key processes in the regulation of homeostasis of adult neurogenesis including the balance between quiescence vs. proliferation, the mode of cell division, and the prevention of stem cell depletion.

The existence of NSCs in the adult neurogenic niches prompted research for their usage in adult brain regeneration. Nevertheless, their intrinsic and extrinsinc properties, which we have summarized above, pose also major challenges to mount adequate therapeutic approaches. MSCs, and specifically the interaction of their properties with NSCs, might be ideal candidates for this purpose. We will next describe the major characteristics of MSCs and how they might promote brain regeneration.

## The Osteogenic Niche

The osteogenic niche is a highly vascularized and dynamic environment in which four cell types play an important role on the maintenance and renewal of bone tissue: MSCs, osteoblasts, osteocytes and osteoclasts.

Osteoblasts (Table 1) arise from osteoprogenitor and MSCs (further details on MSCs biology are discussed in “MSCs and CNS Therapies” Section) present in the bone marrow and periosteum. They are known to be involved in the synthesis and regulation of extracellular matrix elaboration (ECM) and mineralization (Sommerfeldt and Rubin, 2001; Salgado et al., 2004). Furthermore, it is also known that basic cellular functions and responsiveness to metabolic and mechanical stimuli demand are maintained through extensive cell-matrix and cell-cell contacts via a variety of transmembranous proteins and specific receptors (Sommerfeldt and Rubin, 2001). Osteocytes represent osteoblasts that became incorporated in the newly elaborated extracellular matrix, being enclosed in spaces called lacunae. They maintain direct contact with neighboring osteocytes, osteoblasts and bone lining cells through cellular processes that are created before and during matrix synthesis (Sommerfeldt and Rubin, 2001; Knothe et al., 2004). In mature bone these cell processes are contained in channels called the canaliculi. The communication and interaction between neighboring osteocytes is achieved through the establishment of gap junctions (Sommerfeldt and Rubin, 2001; Knothe et al., 2004). This is an absolute need for osteocytes because is the only way by which they can assure the access to oxygen and nutrients. They are known to be involved in the calcification of osteoid matrix, blood-calcium homeostasis and to be the mechanosensor cells of bone (Sikavitsas et al., 2001; Knothe et al., 2004). Finally, **osteoclasts**, are multinucleated polarized cells involved in the bone remodeling process, that belong to the monocyte/macrophage lineage. Their main function is to resorb mineralised bone. For this purpose they are enriched in intracellular structures such as pleomorphic mithocondria, vacuoles, and lysossomes, as well as alterations, namely at the structural level, in its cell membrane (Vaananen, 1996).

## Mesenchymal Stem Cells

### Mesenchymal Stem Cells, The Secretome and Neurogenic Niches

The first reports on the possible existence of a population with a Mesenchymal progenitor character are attributed to Friedenstein et al. (1974b). Indeed, Friedenstein et al. identified and defined these cells as plastic-adherent fibroblast colony-forming units with clonogenic capacity (Friedenstein et al., 1974a). Later, these cells were also named as marrow “stromal cells”, on the basis of their possible use as a feeder layer for hematopoietic stem cells (Eaves et al., 1991; Glavaski-Joksimovic and Bohn, 2013). Additionally other reports also referred to them as MSCs because of their clonogenicity capacity and ability to undergo multilineage differentiation (Caplan, 1991; Bluguermann et al., 2013). Currently MSCs have been defined, according with the International Society for Cellular Therapy (ISCT) criteria, as multipotent cells (with the ability of at least differentiating towards the osteogenic, chondrogenic and adipogenic lineages), capable of self-renewal, able to adhere to tissue culture plastic and to display the presence of surface markers (CD105, CD73, CD90), as well as the lack of hematopoietic cell surface markers (CD45, CD34, CD14 or CD11b, CD79a or CD19 and Human Leukocyte Antigen DR; Table 1; Dominici et al., 2006). So far, MSCs have been isolated from bone marrow (BMSCs), adipose tissue (ASCs), dental pulp, placenta, amniotic fluid, umbilical cord blood, umbilical cord Wharton’s jelly (bulk—WJ-MSCs; perivascular region—human umbilical cord perivascular cells, HUCPVCs), liver, lung and spleen, and brain (for an extensive review see Teixeira et al., 2013). As potential therapeutic agents, MSCs display a number of key characteristics that are believed to be advantageous when compared to other cell populations. For instance they can be isolated with minimal invasive procedures, easily cultured and expanded *in vitro* for several passages, can be used for allogenous transplantation in virtue of their hypoimmunogenicity, decreased tumorigenic potential and, as adult cells, are not hindered by ethical concerns (Salgado et al., 2006; Kishk and Abokrysha, 2011; Seo and Cho, 2012; Teixeira et al., 2013). These MSCs features have made them attractive tools for CNS neurodegenerative diseases.

Initially it was considered that the true therapeutic potential of these cells relied on their multilineage differentiation. Indeed most of the literature of the 90 s and early 21st century was focused on the differentiation of these cells towards mesodermal lineages, such as the osteogenic, mainly within 3D matrices known as scaffolds, to induce regeneration in the affected areas. Around the same time it was also suggested that MSCs even had a greater differentiation potential than was originally predicted, as several reports indicated that these cells could be differentiated beyond the mesodermal lineages (Dominici et al., 2006). In 2005, Gnecchi et al. (2005) put forward a new concept that lately would change the paradigm of how MSCs could be used in regenerative medicine, by showing that their therapeutic potential was mostly related to the growth factors that they secreted to the extracellular milieu, rather than to their differentiation potential.

Indeed, in recent years it is becoming increasingly accepted that the regenerative effects promoted by MSCs are mainly associated with their secretome. As discussed by Teixeira et al. (Teixeira et al., 2013) the secretome of MSCs is composed by a proteic soluble fraction, constituted by growth factors and cytokines, and a vesicular fraction composed by microvesicles and exosomes, which are involved in the transference of proteins and genetic material (e.g., miRNA) to other cells. The protective actions promoted by MSCs secreted molecules are closely related with therapeutic plasticity in the CNS. Indeed several authors have reported the presence of a plethora of growth factors with a known influence on neuronal survival, differentiation, neurite outgrowth and immunomodulation of microglial cells; these factors are BDNF, glial derived neurotrophic factor (GDNF), nerve growth factor (NGF), hepatocyte growth factor (HGF), vascular endothelial growth factor (VEGF), VEGF-receptor 3 (VEGF-R3), angiopoietin 1, insulin-like growth factor 1 (IGF-1), insulin-like growth factor 2 (IGF-2), epidermal growth facto (EGF), basic fibroblast growth factor (bFGF), FGF 20, granulocyte colony-stimulating factor (G-CSF), platelet-derived growth factor AA (PDGF-AA), chemokine ligand 16 (CXCL 16), neutrophil-activating-protein-2 (NAP 2) and neurotrophin-3 (NT-3) growth factors, as well as interleukin-6 (IL-6), interleukin-10 (IL-10), transforming growth factor beta 1 (TGF β1), stem cell factor (SCF), stromal cell-derived factor 1 (SDF-1) and monocyte chemotactic protein 1 (MCP-1) cytokines (Rehman et al., 2004; Caplan and Dennis, 2006; Chen et al., 2008b; Bonfield et al., 2010; Meyerrose et al., 2010; Nakano et al., 2010; Ribeiro et al., 2012). Other proteins such as 14-3-3, ubiquitin carboxyl-terminal esterase L1 (UCHL1), hsp70 and peroxiredoxin-6 have also been related to the neuroregulatory character of the secretome of MSCs (Fraga et al., 2013).

**Figure 2:**
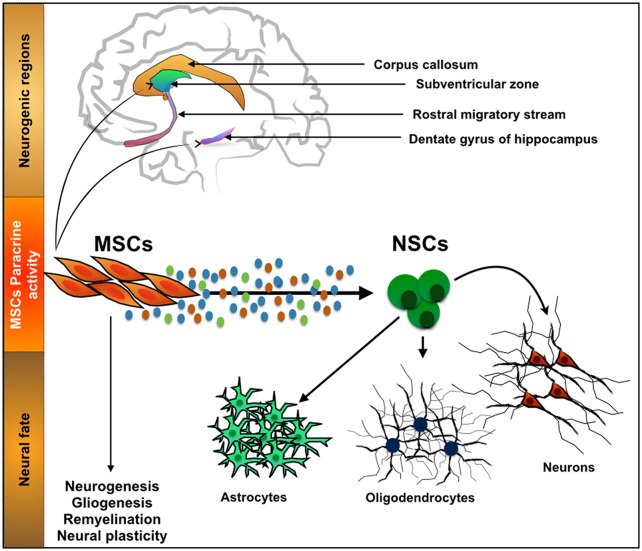
**Interaction between mesenchymal stem cells (MSCs) and neurogenic niches**. MSCs, a cell population with a known function in the osteogenic niche, is able to modulate the action of Neural stem cells (NSCs) by means of their secretome. Through the secretion of neuroregulatory molecules, either soluble or in the form of vesicles, MSCs are able to influence processes such as neurogenesis, gliogenesis, remyelination and neural plasticity. With it important developments have been recently witnessed in CNS regenerative medicine strategies.

The action of MSCs and their secretome in neurogenic niches (Figure 2) such as the SGZ has been previously described. For instance, Munoz et al. (2006) transplanted BMSCs into the DG of immunodeficient mice. Results revealed that the transplanted MSCs markedly increased the proliferation of endogenous NSCs that expressed the stem cell marker Sox2, as well as their differentiation, a fact that was attributed to a local increase on the expression of growth factors such as VEGF, ciliary neurotrophic factor (CNTF), neurotrophin-4/5 and NGF. More recently it was also shown that the injection of the secretome of MSCs itself, was also able to modulate both neuronal survival and differentiation within the adult rat hippocampus. Teixeira et al. (2015) show that the injection of the secretome of HUCPVCs (a MSC population that resides in the perivascular region of the umbilical cord) was able to induce an increased number of DCX^+^ cells. This observation was then related with a higher expression of FGF-2 and NGF in the injected area.

As a consequence of this, the multiple faces of MSCs and their secretome have prompted a number of different experimental therapeutic strategies in CNS regenerative medicine. Such strategies rely on a strong interplay between neuroregulatory molecules secreted by MSCs and the different niches with the CNS.

In disorders such as multiple sclerosis (MS), available data, both from animal models and human patient related studies, indicates that the immunomodulatory properties of the secretome of MSCs regulate the immune/oligodendrogenic niches. For instance Wang et al. (2010) revealed that MSCs derived from human embryonic stem cells (hES-MSCs) significantly reduce clinical symptoms and prevent neuronal demyelination in a mouse experimental autoimmune encephalitis (EAE) mouse model of MS, by reducing the frequency of CD4+ and CD8+ cells infiltration in the CNS. A similar trend was described by Li et al. (2014) and Llufriu et al. (2014) in studies with human patients, in which the administration of MSCs from different sources, alone or combined with pharmacotherapies, positively impacted the condition of the patients, by modulating MS related inflammatory events.

On other hand, in disorders such as PD, Ischemic Stroke (IS) and Glioblastoma Multiforme (GBM) it is believed that the action of MSCs goes beyond the neuro-immunomodulation, and in fact, some of the reported benefits may be closely related with their direct interaction with the neurogenic niches. Due to the nature and objectives of this review, this topic will be further explored in the following section.

### MSCs and CNS Therapies

#### Parkinson’s Disease

Among CNS disorders, PD is the most common motor-related disorder in middle or late-life affecting millions (Pereira and Aziz, 2006) worldwide. It is a slowly progressive neurodegenerative disease that is primarily characterized by the loss of dopaminergic (DAergic) neurons in several dopaminergic networks, most intensively in the ventral tier of the substantia nigra *pars compacta* (SNpc) within the mesostriatal/nigrostriatal pathway (Koller, 2003; Pereira and Aziz, 2006; Cummins and Barker, 2012; Teixeira et al., 2013). The depletion of SN neurons leads to the loss of DAergic innervations and consequently to striatal DA deficiency, which is responsible for the major sensory-motor symptoms of PD (Dauer and Przedborski, 2003).

A considerable body of evidence has revealed the potential of MSCs to promote protection and/or recovery of DAergic neurons against neurotoxin-induced nigrostriatal degeneration. Indeed, several studies have demonstrated that BMSCs secretome protect and/or regenerate DAergic neurons in *in vitro* and *in vivo* models of PD, through the secretion of growth factors and cytokines (summarized in Table 2; Weiss et al., 2006; Shintani et al., 2007; McCoy et al., 2008; Kim et al., 2009; Sadan et al., 2009; Blandini et al., 2010; Cova et al., 2010; Wang et al., 2010; Danielyan et al., 2011; Park et al., 2012). For instance, Shintani and coworkers demonstrated that BMSCs conditioned media (CM) was able to promote survival of tyrosine hydroxylase (TH)-positive DAergic neurons in rat primary cultures of ventral mesencephalic cells (Shintani et al., 2007). Moreover, intrastriatal transplantation of fetal mesencephalic cells treated with human BMSCs CM, during steps of donor preparation and implantation, induced survival of DAergic grafted cells and promoted functional recovery in a 6-OHDA rat model of PD (Shintani et al., 2007). The observed protection of DAergic neurons was attributed to BMSCs secretion of BDNF, GDNF and bFGF. Similarly, Sadan et al. showed that human BMSCs (hBMSCs) cultured in the presence of growth factors, not only significantly increased the viability of the SH-SY5Y neuroblastoma cell line exposed to 6-OHDA, but also that BMSCs transplanted into the striatum of a 6-OHDA rat model of PD, migrated to the lesion site, and increased the numbers of TH-positive cells and DA levels (Sadan et al., 2009). These neuroprotective and neuro-regenerative effects were accompanied by an improvement in animals’ motor behavior and were correlated with BMSCs secretion of BDNF and GDNF. This expression pattern is in accordance with data published by Blandini and co-workers using the same animal model (Blandini et al., 2010). On the other hand, Wang and colleagues associated rat-derived BMSCs expression of stromal cell-derived factor 1 (SDF)-1α with the DAergic neurons protection against 6-OHDA neurotoxin both *in vitro* and *in vivo*, through anti-apoptotic based mechanisms (Wang et al., 2010). Moreover, Cova et al. demonstrated that BMSCs transplanted in the striatum of a 6-OHDA rodent model of PD were able to survive and interact with the lesion site surroundings, thus enhancing the survival of DAergic terminals and neurogenesis in the SVZ in a sustained manner (Cova et al., 2010). Finally, the secretion of BDNF *in vivo* by BMSCs, was correlated with the activation of endogenous stem cells (Cova et al., 2010).

**Table 2:** **Summary of the studies focused on the impact of MSCs on multiple aspects of PD regenerative medicine**.

Reference	Outcomes
Weiss et al. (2006)	• Transplantation of MSCs isolated from the Wharton Jelly into a 6-OHDA rat model led to behavioral improvements and a local increase of GDNF.
Shintani et al. (2007)	• CM of BMSCs promoted survival of TH^+^ neurons; • Transplantation of fetal mesencephalic cells treated with BMSCs CM promoted functional recovery in a 6-OHDA rat model.
Sadan et al. (2009)	• BMSCs transplantation into a 6-OHDA rat model led to increased TH+ cells and tissue DA levels;
	• Data correlated with secretion of GDNF by BMSCs.
Wang et al. (2010)	• BMSCs protected DA neuronal apoptotic cell death through SDF-1α.
Cova et al. (2010)	• Long term survival of BMSCs upon transplantation into the striatum;
	• Increased neurogenesis in SVZ;
	• Survival of DAergic terminal.
Danielyan et al. (2011)	• Intranasal delivery of BMSCs in a 6-OHDA rat model reduced the levels of pro-inflammatory cytokines.

*OHDA, Hydroxidopamine; GDNF, Glial Derived Neurotrophic Factor; TH, Tyrosine Hydroxylase; CM, Conditioned Media; DA, Dopamine; SDF, Stromal Cell Derived Factor; SVZ, Subventricular Zone*.

In addition to the capability of BMSCs to induce survival of DAergic neurons, its effects have also been related with their immunomodulatory properties. In this context, intranasally delivered rat BMSCs into 6-OHDA hemi-parkinsonian rats migrated toward the SN and the striatum and reduced the overall expression of pro-inflammatory cytokines, such as IL-1β, IL-2; IL-12; tumor necrosis factor alpha (TNF-α) and interferon γ (INF γ). Moreover, their presence also revert the loss of nigral DAergic neurons and striatal fibers (Danielyan et al., 2011).

From the above-referred studies, it is clear that there is increasing evidence indicating that the neuroprotective and neuroregenerative effects of MSCs observed in PD are attributed to the secretion of soluble growth factors and cytokines. The secretion of these factors by MSCs not only protects DAergic neurons from further degeneration and enhances endogenous restorative processes (e.g., neurogenesis), but also acts as inflammation and immune response modulators. Moreover, recent reports have shown that besides soluble growth factors and cytokines, MSCs also secrete microvesicles and exosomes containing mRNAs and/or miRNAs (microRNAs), which are believed to mediate cell-to-cell communication and act as reparative agents (Baglio et al., 2012). Indeed exosomes secreted by BMSCs *in vitro* not only mediate communication with neurons and astrocytes, but also regulate neurite outgrowth by transfer of miRNA (miR-133b) to neural cells (Xin et al., 2012).

#### Ischemic Stroke (IS)

Cerebrovascular diseases, such as stroke, result from blood vessel occlusion or damage, leading to focal tissue loss and death of endothelial cells and multiple neural populations (Lindvall and Björklund, 2004; Lindvall and Kokaia, 2010).

It has been proposed that the transplantation of MSCs (summarized in Table 3) can represent a feasible therapeutic option for IS (Locatelli et al., 2009). Indeed, studies have shown that after intravenous administration of BM-MSCs, these have the capacity to migrate to the lesion site promoting tissue regeneration and behavioral improvement (Komatsu et al., 2010). Moreover, these cells were able to promote neurogenesis, increase the survival of neuroblasts and to reduce the volume of lesion after IS (Keimpema et al., 2009; Zheng et al., 2010). According to Wakabayashi and colleagues the secretion of molecules such as IGF-1, VEGF, EGF, BNDF and bFGF mediate some of the observed effects, namely the reduction of lesion size and the modulation of the inflammatory environment for host cells (Wakabayashi et al., 2010). Leu et al. (2010) also proposed that like BM-MSCs, adipose stroma/stem cells (ASCs) therapy also enhances angiogenic and neurogenic processes. Although the exact mechanism of these cells remains still unclear, other studies have suggested that homing properties, cytokines (SDF-1α, IL-1, IL-8) effects, and paracrine mediators (HGF, BDNF, IGF-1, VEGF) could pinpoint ASCs effects, contributing to tissue regeneration and functional behavior (Tang et al., 2005; Banas et al., 2008; Chen et al., 2008a). On the other hand Koh et al. (2008) also demonstrated that MSCs exhibited a migratory tropism to the lesion site, which might foster the creation of new networks between the host neural and transplanted stem cells (Koh et al., 2008). Additionally exosomes secreted by MSCs were also shown to mediate important actions in these environments. Xin et al. (2013b) suggested that the observed improvements were due to the presence of miRNA-133b in the exosomal fraction of MSCs that were transplanted into a middle cerebral artery occlusion (MCAo) rat model. Similarly, the same authors also demonstrated that after systemic administration of MSCs-derived exosomes, there was an increase in neurovascular plasticity, which led to an enhancement of the functional recovery of an animal model of stroke (Xin et al., 2013a,b).

**Table 3:** **Impact of MSCs administration on ischemic stroke related animal models**.

**Reference**	**Outcomes**
Koh et al. (2008)	• MSCs exhibited migratory tropism to injury sites.
Komatsu et al. (2010)	• Intravenous delivery of MSCs promoted tissue regeneration and behavioral improvement.
Keimpema et al. (2009);	• Reduction of the volume of the injury after IS;
Zheng et al., 2010	• Increased levels of neurogenesis;
	• Survival of neuroblasts.
Wakabayashi et al. (2010)	• Reduction of the injury size and modulation of the inflammatory environment through the secretion of IGF-1, VEGF, EGF, BNDF and bFGF.
Leu et al. (2010)	• ASCs based therapies enhanced angiogenic and neurogenic processes in IS models.
Xin et al. (2013a)	• Systemic administration of exosomal fraction of the secretome impacted neurovascular plasticity.

*IGF, Insulin Growth Factor; VEGF, Vascular Endothelial Growth Factor; EGF, Epidermal Growth Factor; BDNF, Brain Derived Neurotrophic Factor; bFGF, basic Fibroblast Growth Factor; ASCs, Adipose Tissue Stem Cells*.

#### Glioblastoma Multiforme (GBM)

Malignant gliomas are particularly dramatic cancers of the CNS, ranking first among all human tumor types for tumor-related average years of life lost (Burnet et al., 2005). GBM is the most common and most malignant subtype (Ohgaki and Kleihues, 2007), typically treated with surgery, radiotherapy and temozolomide (TMZ)-based chemotherapy (Stupp et al., 2005). Despite this multimodal approach, virtually all GBMs eventually recur and are fatal. GBMs present critical hallmark features that largely contribute to treatment failure, including their high invasive capacity, the presence of the bood-brain barrier, and remarkable genetic and epigenetic heterogeneity. Additionally, GBMs present a small population of cells with neural stem cell-like properties (Singh et al., 2003), called glioma stem cells (GSC), which display remarkable features in the context of glioma pathophysiology, including self-renewal capacity (generating both GSCs and non-GSCs cancer cells necessary for tumor maintenance), multipotency (differentiating into diverse cell population lineages), and prominent tumorigenic potential *in vivo*. In resemblance with NSCs that are located in specific highly-vascularized neurogenic niches of the adult brain, GSCs also accumulate and depend on the prominent vasculature of these regions to control their stemness and differentiation processes (Folkins et al., 2007; Calabrese et al., 2007; Gilbertson and Rich, 2007; Hadjipanayis and van Meir, 2009). GSCs have been shown to be more resistant to radiation and conventional chemotherapeutic drugs, and are believed to be responsible for tumor relapse observed almost universally in GBM patients (Singh et al., 2003; Bao et al., 2006; Calabrese et al., 2007; Chalmers, 2007). Since the clinical prognosis of GBM patients has not improved significantly in the last years, it is urgent to develop novel unconventional therapeutic strategies.

Like in other cancer types, a relatively new and promising therapeutic approach to tackle malignant gliomas is based on the use of (normal) stem cells. The most unique and critical feature of stem cells that renders them as attractive tools for cancer therapy is their intrinsic capacity to migrate towards pathologic tissues, including malignant tumors. Indeed, this selective cancer-tropism has been shown for various stem cell types, including embryonic, hematopoietic, mesenchymal, neural, endothelial, and experimentally-induced stem cells (e.g., inducible pluripotent stem cells, iPSCs; Stuckey and Shah, 2014). Whether this innate tropism of normal stem cells is associated with cancer promotion or suppression functions is still controversial and a matter of debate, particularly in the case of MSCs, as reported by contradicting findings in many studies (Klopp et al., 2011). Nonetheless, it is widely consensual that the rational engineering of stem cells to express or deliver anticancer therapeutic agents, while taking advantage of their innate tumor tropism and immunosuppressive properties, may be a promising strategy to target cancer.

Aboody et al. (2000) first showed that NSCs are able to migrate towards the major tumor site and track along with invading glioma cells that form small satellite tumor masses (Aboody et al., 2000). Importantly, this tumor-tropism by NSCs was also later observed towards brain metastasis derived from breast cancer (Joo et al., 2009) and melanoma (Aboody et al., 2006), highlighting the potential application of NSCs as therapeutic vehicles for primary and metastatic brain tumors. In this context, and because stem cells are relatively easy to be genetically modified, many studies have explored them as cargo delivery vehicles for therapeutic agents, including cytokines, pro-drug converting enzymes, oncolytic viruses, nanoparticles, and antibodies, as summarized below.

##### Cytokines

Many recent studies have explored NSCs as efficient delivery systems of soluble tumor necrosis factor-related apoptosis-inducing ligand (sTRAIL), a cytokine that promotes apoptosis by binding to death receptors commonly present in the cellular membrane of tumor cells. These engineered NSCs can track tumor cells and deliver sTRAIL to glioma cells *in vivo*, resulting in significant anti-tumor effects. Combinations of sTRAIL-secreting NSCs with anticancer drugs, including bortezomib (a proteasome inhibitor), PI-103 (a dual PI3K/mTOR inhibitor), and lanatoside C (a cardiac glycoside), resulted in synergistic therapeutic effects, emphasizing the potential clinical value of sensitizing glioma cells to TRAIL-induced NSC-mediated cell death (Hingtgen et al., 2010; Bagci-Onder et al., 2011; Balyasnikova et al., 2011; Teng et al., 2014). Importantly, studies with MSCs engineered to deliver sTRAIL showed equally promising results, as these cells efficiently tracked and successfully induced a caspase-dependent cell death in glioma cells, resulting in increased survival of glioma mice models (Shah et al., 2004; Menon et al., 2009; Sasportas et al., 2009; Choi et al., 2011).

NSCs have also been genetically modified to express and secrete IL-12, a cytokine that does not act directly in tumor cells, but is involved in the enhancement of T-cell-mediated antitumor immune responses. Using intracranial glioma mice models, Ehtesham et al. showed that IL-12-secreting NSCs injected directly in the tumor significantly prolong the survival of mice (Ehtesham et al., 2002). Similarly, MSCs genetically engineered to express a modified IL-12 also prolonged the survival of glioma mice models when injected intratumorally (Ryu et al., 2011). Similar approaches were used to engineer NSCs, MSCs, and bone marrow-derived stem cells to produce pro-inflammatory cytokines, including IL-4, IL-7, IL-23, and IFN-β, which were shown to increase the infiltration of anti-tumor T-cells and natural killer (NK)-cells in glioma murine models (Benedetti et al., 2000; Nakamizo et al., 2005; Yuan et al., 2006; Gunnarsson et al., 2010). These studies provide important proof-of-concept on the potential of modulating immune mediators with different types of stem cells in order to achieve increased therapeutic responses.

##### Enzymes/pro-drugs

Another novel approach involves the modification of stem cells to express enzymes that convert inactive pro-drugs into toxic compounds, in order to increase tumor tissue selectivity. One of the most popular pro-drug/enzyme therapeutic systems is the herpes simplex virus type 1 thymidine kinase (HSV-tk) in combination with the pro-drug ganciclovir (GCV), based on the HSV-tk-mediated phosphorylation of inert GCV into a cytotoxic product that kills HSV-tk-positive cells and neighboring cells (via the so-called bystander effect). Taking advantage of the tumor-tropism of stem cells, many recent studies have explored the incorporation of HSV-tk into NSCs, MSCs, and bone marrow-derived progenitor cells as therapeutic strategies for glioma, showing promising results (Li et al., 2005; Uhl et al., 2005; Miletic et al., 2007; Uchibori et al., 2009; Matuskova et al., 2010). Other enzyme/pro-drug systems that have been explored as anti-cancer therapeutic tools for stem cells include the cytosine deaminase (CD), which converts inactive 5-fluorocytosine (5-FC) into the cytotoxic 5-fluorouracil (5-FU), and the rabbit carboxylesterase enzyme (rCE), which converts the pro-drug CTP-11 (irinotecan) into the anticancer topoisomerase I inhibitor SN-38 (7-ethyl-10-hydroxycamptothecin). These approaches have been tested with promising therapeutic results in stem cells of different origin (NSCs and MSCs) and distinct glioma models (including rat and mice models), either alone or in combination with other anticancer drugs (Aboody et al., 2000, 2006; Lim et al., 2011; Yin et al., 2011; Choi et al., 2012; Fei et al., 2012; Kim et al., 2012; Kosaka et al., 2012; Ryu et al., 2012; Zhao et al., 2012), emphasizing the potential of these enzyme/pro-drug systems as stem cell-mediated anti-tumor therapies.

##### Oncolytic viruses

The use of oncolytic viruses as therapeutic agents has been extensively studied for cancer, taking advantage of their capacity to infect, replicate within, and ultimately kill cancer cells. Despite many promising pre-clinical studies, including in gliomas (Wollmann et al., 2012), the clinical application of oncolytic viruses presents critical obstacles, including sub-optimal distribution throughout the major tumor cores and particularly to invading cancer cells, low infection rates, and host anti-viral immune responses (Yamamoto and Curiel, 2010). Critically, these shortcomings can be largely surpassed by the incorporation of oncolytic viruses within tumor-trophic stem cells. Indeed, recent work has been performed in NSCs, MSCs and ASCs that were used as oncolytic viral carriers to treat *in vivo* models of glioma, showing that these cells retain tumor-tropism, permit continued viral replication for several days, and cause glioma cell death *in vivo* more efficiently than viral delivery alone (Herrlinger et al., 2000; Sonabend et al., 2008; Tyler et al., 2009; Yong et al., 2009; Josiah et al., 2010; Ahmed et al., 2011; Thaci et al., 2012).

##### Nanoparticles and antibodies

In the last 4 years, some studies also started to explore MSCs as delivery vehicles of drug-loaded nanoparticles and antibodies to target glioma. This strategy aims to improve the capacity of these agents to cross the blood-brain barrier, while minimizing toxic side effects caused by intravenous administrations. The results obtained to date indicate that these cells can successfully deliver nanoparticles (e.g., lipid nanocapsules loaded with ferrociphenol and membrane-anchored silica nanorattle–doxorubicin) and antibodies (e.g., cell surface-bound single-chain anti-EGFRvIII) to glioma cells *in vivo*, resulting in increased anti-tumor responses (Balyasnikova et al., 2010; Roger et al., 2010, 2012; Li et al., 2011).

In conclusion, a wide variety of stem cells hold great promise as novel therapeutic tools for the treatment of therapy-insensitive malignant brain gliomas. Some hallmarks of these cells that are critical for this purpose include their high tumor-trophic migration and tracking capacity, peculiar immunosuppressive properties, and easy genetic manipulation for cargo delivery. Nonetheless, inherently to its innovative nature and similarly to other experimental glioma therapies attempted in the past, several issues will certainly need to be addressed in order to translate these promising pre-clinical findings into clinically-relevant therapies for patients. Some of the obstacles that may be envisaged include the proper selection of the best stem cell type/origin, choice of the most appropriate cargo for each tumor type or personalized to specific patients, optimization of administration routes and dosing, evaluation of the long-term cell fate of engrafted stem cells (which may conceptually also form tumors or differentiate aberrantly in the target tissue/organ), and development of real-time imaging systems for therapeutic stem cells *in vivo*. The recent literature on this topic is very promising, but a concerted and integrated effort in this field will still be crucial to definitely pave the way to better treat patients, most likely integrating the rational use of particular stem cell-based approaches to act synergistically in concert with surgery, radiation and chemotherapy.

## Conclusion

It is now evident that cells derived from the osteogenic and neurogenic niches present important interactions that may impact the future development of CNS related therapies. As discussed in the present review there is robust evidence showing that MSCs and their secretome are able to modulate the action of neurogenic niches and neural progenitors. Their usage was shown to promote the functional recovery of animal models of PD and stroke, as well as the application of novel paradigms for glioblastoma therapies. Nevertheless, it is still a largely unexplored field, with many questions yet to be addressed. For instance, are the traditional growth factors the main mediators of the actions promoted by the MSCs secretome; or, instead, do MSCs-derived unknown neuroregulatory molecules modulate such actions? Can we modulate the tropism that these cells display towards gliobastomas? So far, most of the studies focused on the action of MSCs towards the neurogenic niches, namely NSCs. However, few address if and how the neurogenic niches, and within them NSCs, modulate the action of MSCs. In fact a bidirectional communication between both cell types is most likely to occur. The answer to this and other questions will be important to further define this field in the future, and its impact in future CNS regenerative strategies.

## Conflict of Interest Statement

The authors declare that the research was conducted in the absence of any commercial or financial relationships that could be construed as a potential conflict of interest.
